# Single-Step Genome Editing of Small Ruminant Embryos by Electroporation

**DOI:** 10.3390/ijms231810218

**Published:** 2022-09-06

**Authors:** Ahmed K. Mahdi, Juan F. Medrano, Pablo J. Ross

**Affiliations:** Department of Animal Science, University of California, Davis, CA 95616, USA

**Keywords:** genome editing, CRISPR-Cas9, electroporation, single step, small ruminants

## Abstract

We investigated the possibility of single-step genome editing in small ruminants by CRISPR-Cas9 zygote electroporation. We targeted SOCS2 and PDX1 in sheep embryos and OTX2 in goat embryos, utilizing a dual sgRNA approach. Gene editing efficiency was compared between microinjection and three different electroporation settings performed at four different times of embryo development. Electroporation of sheep zygotes 6 h after fertilization with settings that included short high-voltage (poring) and long low-voltage (transfer) pulses was efficient at producing SOCS2 knock-out blastocysts. The mutation rate after CRISPR/Cas9 electroporation was 95.6% ± 8%, including 95.4% ± 9% biallelic mutations; which compared favorably to 82.3% ± 8% and 25% ± 10%, respectively, when using microinjection. We also successfully disrupted the PDX1 gene in sheep and the OTX2 gene in goat embryos. The biallelic mutation rate was 81 ± 5% for PDX1 and 85% ± 6% for OTX2. In conclusion, using single-step CRISPR-Cas9 zygote electroporation, we successfully introduced biallelic deletions in the genome of small ruminant embryos.

## 1. Introduction

Small ruminants are important animals in agricultural systems and biomedical research. Their size, anatomy, and gestation period make them appropriate for sustainable food production, human diseases investigations, and xenotransplantation research [1]. The CRISPR-Cas9 system is a powerful genome editor that can modify specific genomic sequences in a simple and efficient manner [2,3]. Since first introduced, the CRISPR/Cas9 system has continued to be optimized and has proven to be useful for multiple purposes [4]. In small ruminants, the CRISPR-Cas9 approach was used to produce genome-edited knock-out [5] and knock-in [6] animals.

There are two general approaches to producing genome-edited animals, the single-step approach and the two-step approach. In the single-step approach, editing occurs directly in zygotes, which are then transferred to surrogates to generate a gene-edited animal. In the two-step approach, gene edits are introduced into cultured cells, and then gene-edited cells are used to create an animal by somatic cell nuclear transfer (SCNT)/cloning. In general, because of the technical difficulty and the low efficiency of SCNT/cloning [7], the single-step approach would be advantageous.

When producing genome-edited animals in a single-step approach, the CRISPR-Cas9 system is delivered directly to the zygotic cytoplasm. Microinjection is an effective method for delivering genome editing reagents to a zygote. However, microinjection requires expensive equipment and intensive training. In addition, the microinjection process is time-consuming, and results can be inconsistent, especially when processing a large number of zygotes. Moreover, mosaicism has been repeatedly reported in animals developed from microinjection of engineered endonucleases to zygotes, hindering the benefit of the single-step CRISPR-Cas9 strategy [8]. The alternative method of delivering the CRISPR-Cas9 system to zygotes is electroporating zygotes to generate resealable pores in the plasma membrane and facilitate the passive diffusion of genome editor components to the pronuclei. Many approaches have been used to deliver the CRISPR-Cas9 system efficiently to the mammalian zygotes by electroporation, such as weakening the zona pellucida (ZP) by acid Tyrode’s solution before electroporation [9]. Lately, successful attempts have been reported to produce CRISPR-Cas9-edited rats [10], mice [11], pigs [12], and cattle [13] by electroporation of ZP-intact zygotes.

This study investigated the efficiency of electroporation as a delivery method of CRISPR-Cas9 components to the sheep and goat zygotes. We targeted two different loci in the ovine genome: SOCS2 (suppressor of cytokine signaling 2) on chromosome 3 and PDX1 (pancreatic and duodenal homeobox protein 1) on ovine chromosome 10, and one locus in the caprine genome: OTX2 (orthodenticle homeobox 2) on chromosome 10. SOCS2 regulates somatic growth by inhibiting the transcription pathways induced by GH, EGF, and IGF-1 [14]. PDX1 is a transcription factor that is essential to pancreas development [15], and OTX2 is a transcription factor that is necessary for head development [16]. 

## 2. Results

### 2.1. CRISPR/Cas9 Electroporation Optimization for Sheep Oocytes/Embryos

To optimize gene editing in sheep zygotes, CRISPR/Cas9 reagents targeting sheep SOCS2 were delivered to sheep oocytes/zygotes using different parameters. Sheep oocytes/zygotes were electroporated using one of three different settings (Table 1). Setting 1 included 4 poring pulses of 3.5 ms at 50 ms intervals, starting at 40 V with a 10% decay rate. Setting 2 was similar, except that the electroporator was set up to also deliver 5 transfer pulses of 50 ms at 50 ms intervals, starting at 5 V with a 40% decay rate followed by another set of transfer pulses in the opposite polarity. Setting 3 included 6 poring pulses of 3 ms at 100 ms intervals and 30 V each, followed by another set of identical pulses in opposite polarity. Electroporation was performed in metaphase II (MII) oocytes and in zygotes at 6, 8, and 10 h post fertilization. Microinjection was performed in MII oocytes 6 h after fertilization.

#### 2.1.1. Effect of Electroporation on Day 7 Embryonic Development

**Figure 1:**
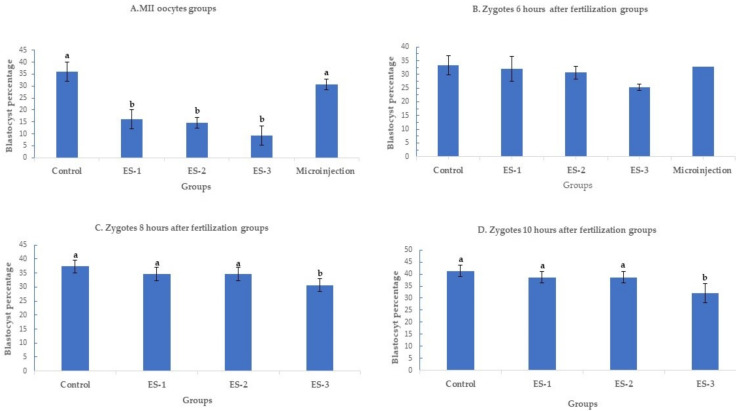
Blastocyst rates after targeting SOCS2 in sheep (means ± standard deviation). Means with different letters differ significantly (*p* < 0.05). Development after microinjection or electroporation in (**A**–**D**) ES-1: Electroporation setting 1 (voltage: 40 V, pulse length: 3.5 ms, pulse interval: 50 ms, pulse number: 4, decay rate 10%, polarity: +). ES-2: Electroporation setting 2 (poring: voltage: 40 V, pulse length: 3.5 ms, pulse interval: 50 ms, pulse number: 4, decay rate 10% polarity: +. Transfer: voltage: 5 V, pulse length: 50 ms, pulse interval: 50 ms, pulse number: 5, decay rate 40%, polarity: +/−). ES-3: Electroporation setting 3 (voltage: 30 V, pulse length: 3 ms, pulse interval: 100 ms, pulse number: 6, polarity: +/−).

Day 7 blastocyst percentages are summarized in Figure 1 (means ± standard deviation). Electroporation of MII oocytes significantly (*p* < 0.01) reduced blastocyst development in an electroporation parameter-dependent manner, with setting 3 showing the lowest developmental rate, 9.3% ± 4% (7/75), compared to 36% ± 4% (27/75) in the control. Conversely, microinjection of MII oocytes had no significant effect on the day 7 development rate. The blastocyst percentages were 16% ± 4% (12/75), 14.6% ± 2% (11/75) and 30.7% ± 2% (23/75) for electroporation setting 1, electroporation setting 2 and microinjection (Figure 1A). Notably, electroporation of zygotes 6 h after fertilization had no significant (*p* > 0.05) effect on day 7 development rate. The blastocyst percentage was 33.3% ± 4% (25/75) in the control group, 30.6% ± 3% (23/75) in electroporation setting 1 group, 30.6% ± 4% (23/75) in electroporation setting 2 group, 25.3% ± 2% (19/75) in electroporation setting 3 group, and 32% ± 1% (24/75) in microinjection group (Figure 1B). Similarly, electroporation of zygotes 8 and 10 h after fertilization did not demonstrate any significant (*p* > 0.05) effect on blastocyst rate except for the electroporation setting 3 group. When the electroporation was performed 8 h after fertilization, the blastocyst percentages were 37.3% ± 2% (28/75), 34.6% ± 2% (26/75), 34.6% ± 2% (26/75), and 30.6% ± 2% (23/75) in the control, electroporation setting 1, 2, and 3 groups, respectively (Figure 1C). While the blastocyst percentages were 41.3% ± 2% (31/75), 38.6% ± 2% (29/75), 38.6% ± 2% (29/75), and 32% ± 4% (24/75) in the control, electroporation setting 1, 2, and 3 groups (Figure 1D).

#### 2.1.2. Effect of Electroporation on SOCS2 Mutation Rates

**Figure 2:**
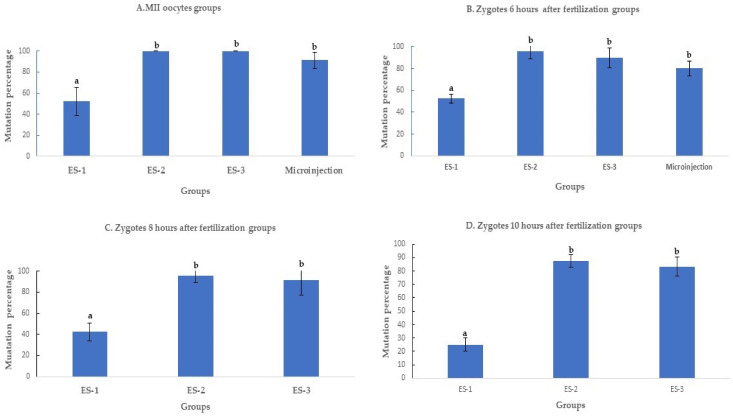
Mutation rates after targeting SOCS2 in sheep (means ± standard deviation). Means with different letters differ significantly (*p* < 0.01). Mutation rate after microinjection or electroporation in (**A**–**D**). ES-1: Electroporation setting 1 (voltage: 40 V, pulse length: 3.5 ms, pulse interval: 50 ms, pulse number: 4, decay rate 10%, polarity: +). ES-2: Electroporation setting 2 (poring: voltage: 40 V, pulse length: 3.5 ms, pulse interval: 50 ms, pulse number: 4, decay rate 10% polarity: +. Transfer: voltage: 5 V, pulse length: 50 ms, pulse interval: 50 ms, pulse number: 5, decay rate 40%, polarity: +/−). ES-3: Electroporation setting 3 (voltage: 30 V, pulse length: 3 ms, pulse interval: 100 ms, pulse number: 6, polarity: +/−).

Electroporation of the CRISPR-Cas9 system increased the rates of mutation when compared to microinjection. The improvement was time and electroporation parameters dependent. All the electroporated MII oocytes were mutant when electroporated using settings 2 and 3. The mutation percentages with setting 2 (100% ± 0%, 11/11), setting 3 (100% ± 0%, 7/7), and microinjection (91.3% ± 8%, 21/23) were superior (*p* < 0.01) to electroporation setting 1 (50% ± 16%, 6/12) (Figure 2A). Likewise, the mutation percentage of setting 2 (95.6%± 8%, 22/23), setting 3 (89.4% ± 10%, 17/19), and microinjection (83.3% ± 8%, 20/24) were higher (*p* < 0.01) than in setting 1 (52.1% ± 4%, 12/23), when the delivery of the CRISPR-Cas9 system was performed 6 h after fertilization (Figure 2B).

**Figure 3:**
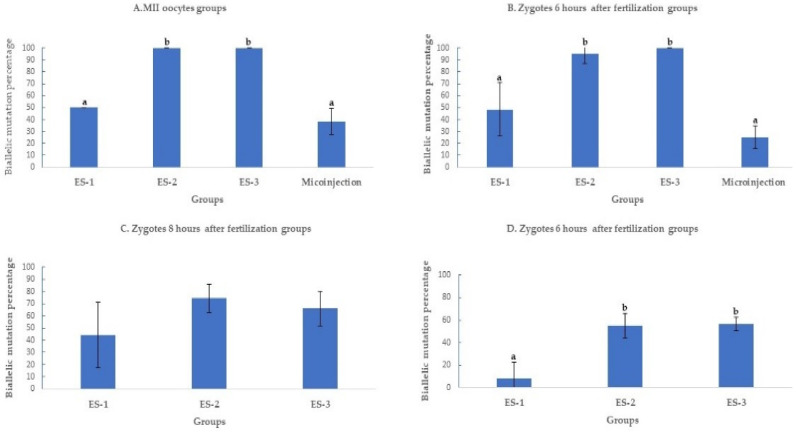
Biallelic mutation rate after targeting SOCS2 in sheep (means ± standard deviation). Means with different letters differ significantly (*p* < 0.01). Biallelic mutation rates after microinjection or electroporation in (**A**–**D**). ES-1: Electroporation setting 1 (voltage: 40 V, pulse length: 3.5 ms, pulse interval: 50 ms, pulse number: 4, decay rate 10%, polarity: +). ES-2: Electroporation setting 2 (poring: voltage: 40 V, pulse length: 3.5 ms, pulse interval: 50 ms, pulse number: 4, decay rate 10% polarity: +. Transfer: voltage: 5 V, pulse length: 50 ms, pulse interval: 50 ms, pulse number: 5, decay rate 40%, polarity: +/−). ES-3: Electroporation setting 3 (voltage: 30 V, pulse length: 3 ms, pulse interval: 100 ms, pulse number: 6, polarity: +/−).

When the electroporation was executed 8 and 10 h after fertilization (Figure 3C,D), the mutation percentage decreased from 42.3% ± 7% (11/26) to 31% ± 7% (9/29) in setting 1, from 96% ± 7% (25/26) to 86.2% ± 10% (25/29) in setting 2, and from 91.3% ± 13% (21/23) to 83.3% ± 8% (20/24) in setting 3. The mutation percentages in setting 2 and setting 3 of electroporation were significantly higher (*p* < 0.01) than in setting 1 (Figure 2C,D).

#### 2.1.3. Electroporation Increased SOCS2 Biallelic Mutation Rate

The biallelic mutation percentage was significantly (*p* < 0.01) higher when MII oocytes were electroporated using settings 2 (100%;11/11) and 3 (100%; 7/7), compared to setting (50%; 3/6) 1 and microinjection (38.1%; 8/21) (Figure 3A). At 6 h post fertilization, the biallelic mutation rate decreased to 25% ± 10% (5/20) in microinjection compared to 95.4% ± 9% (21/22) and 100% ± 0% (17/17) in settings 2 and 3 of electroporation (Figure 3B). Past 6 h of fertilization, the biallelic mutation rates continued to decrease as time post fertilization increased. At 8 h of fertilization, the biallelic mutation rates were 45.4% ± 27% (5/11), 76% ± 12% (19/25), and 66.6% ± 14% (14/21) for settings 1, 2, and 3 of electroporation (Figure 3C) further decreasing at 10 h of fertilization to 11.1% ± 11% (1/9), 56% ± 12% (14/25), and 60% ± 5% (12/20) in settings 1, 2, and 3 of electroporation (Figure 3D).

#### 2.1.4. Targeting an Alternative Locus by Zygote Electroporation of CRISPR/Cas9

**Figure 4:**
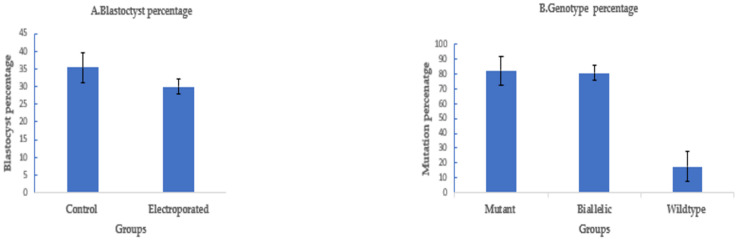
Targeting PDX1 in sheep by CRISPR-Cas9 electroporation (means ± standard deviation). (**A**) Developmental rate. (**B**) Mutation rate. Electroporation parameters (poring: voltage: 40 V, pulse length: 3.5 ms, pulse interval: 50 ms, pulse number: 4, decay rate 10% polarity: +. Transfer: voltage: 5 V, pulse length: 50 ms, pulse interval: 50 ms, pulse number: 5, decay rate 40%, polarity: +/−).

To assess the efficiency of electroporation-based single-step genome editing targeting an alternative locus, we electroporated dual sgRNA CRISPR/Cas9 reagents targeting PDX1. Electroporation, 6 h after fertilization, using setting 2, successfully deleted 208 bp of PDX1 sequence in 37 out of 45 electroporated sheep zygotes (82.2% ± 8%), with 30 out of 37 mutated embryos (81% ± 5%) having a biallelic mutation. The blastocyst rate was 30% ± 2% (45/150) in the electroporated group compared to 35.5% ± 4% (53/150) in the control (Figure 4).

#### 2.1.5. Targeting OTX2 in Goat Oocytes/Embryos by CRISPR/Cas9 Electroporation

**Figure 5:**
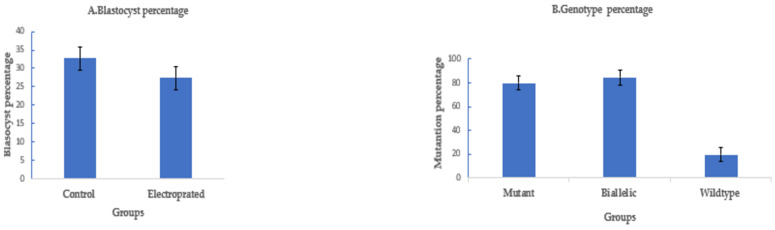
Targeting OTX2 in goats by CRISPR-Cas9 electroporation. (means ± standard deviation). (**A**) Developmental rate. (**B**) Mutation rate. (Poring: voltage: 40 V, pulse length: 3.5 ms, pulse interval: 50 ms, pulse number: 4, decay rate 10% polarity: +. Transfer: voltage: 5 V, pulse length: 50 ms, pulse interval: 50 ms, pulse number: 5, decay rate 40%, polarity: +/−).

To assess if electroporation of CRISPR/Cas9 would also work for goat zygotes, we targeted OTX2 with a dual sgRNA approach. A long deletion of 1.1 kb was successfully introduced in the OTX2 locus of the goat genome by electroporation of zygotes 6 h after fertilization using setting 2. The blastocyst rate was 27.3% ± 3% (41/150) compared to 32.7% ± 3% (49/150) in the control. The mutation percentage was 80.5% ± 5% (33/41), with a biallelic mutation rate of 85% ± 6% (28/33) (Figure 5).

## 3. Discussion

In this study, we produced knock-out blastocysts from two small ruminant species, sheep (*Ovis aries*) and goat (*Capra hircus*), using electroporation to deliver the CRISPR-Cas9 system reagents. Different electroporation settings were used at different times of embryo development to optimize the creation of biallelic mutant (knock-out) embryos. Electroporating sheep zygotes 6 h after in vitro fertilization using a setting consisting of poring and transfer pulses (setting 2) was superior to microinjection in producing more biallelic mutant blastocysts. The mutation percentage increased from 83.3% ± 8% (20/24) to 95.6% ± 8% (22/23) (Figure 3B).

During microinjection, a single opening is usually made in the plasma membrane, and the CRISPR-Cas9 RNPs are placed close to the plasma membrane. In electroporation, the poring electric pulses induce millions of small (~1 nm) resealable pores in the plasma membranes [17] for a time window of 6.4–29.5 nanoseconds [18]. Polarity is a well-described feature of mammalian oocyte cytoplasm [19], which may prevent or hinder the RNPs trafficking to the nucleus. The multi-poration of the plasm membrane most likely increases the availability and even accessibility of the RNPs to the genome.

Biallelic mutation rate decreased when the time of CRISPR-Cas9 RNPs delivery was delayed in microinjection from 38% ± 10% (8/21) (MII oocytes) to 25% ± 10% (5/20) (6 h zygotes) and in electroporation from 100% ± 0% (11/11) in MII oocytes to 95.4% ± 9% (21/22) in 6 h zygotes, 76% ± 12% (19/25) in 8-h zygotes and 56% ± 12% (14/25) in 10-h zygotes. The RNPs delivery time is thus of critical importance for genome editing of zygotes. To reduce the chances of mosaicism, DNA editing must be completed before the first DNA replication, which occurs ahead of the first embryonic cleavage. In mice, electroporation of the zygotes 12 h after fertilization increased the rate of mosaicism [20]. In our study, ICE analysis was satisfying to obtain a solid conclusion about the efficiency of electroporation; however, this approach cannot provide a clear indication of the presence of mosaicism or chimeras. Accurate chimerism analysis requires single cell or single molecule sequencing, which was outside the scope of this project. Nevertheless, electroporation of zygotes 8 h after IVF resulted in decreased biallelic mutation percentage, which is an indication of reduced efficiency of CRISPR/Cas9 that could be associated with acting after DNA replication, therefore requiring the mutation of 4 copies of the gene, instead of just 2, and an increase in embryo chimerism.

An experienced technician could process up to 500 zygotes in one hour by electroporation (from stripping the zygotes to the culture of the electroporated embryo) compared to 40–50 zygotes that could be processed by microinjection. Moreover, electroporation maintains consistency across embryos in the dose of RNPs delivered, which is harder to control in microinjection, especially when processing large numbers of zygotes.

Electroporation of the MII oocyte with settings 2 and 3 (Table 1) produced a 100% biallelic mutation rate compared to 38 ± 10% in the microinjection group. However, electroporation of MII oocytes significantly decreased (*p* < 0.01) the developmental rate from 30.7% ± 7% in microinjection to 14.6% ± 2% and 9.3% ± 4% in settings 2 and 3 of electroporation, respectively. Electroporation changes the structure and the components of the plasma membrane. Besides inducing resealable pores in the membrane lipid bilayers, electroporation increases lipid peroxidation [21] and can alter protein structures [22], which may affect fertilization progression, induce block to fertilization, or increase the vulnerability to polyspermy in the electroporated MII oocytes. Further elucidation is needed to clarify the negative influence of oocyte electroporation on embryo development.

Microinjection and electroporation using settings 2 and 3 were superior to electroporation setting 1 (Table 1). The efficiency of electroporation depends on the electroporation parameters [23,24]. Pore number is correlated with pulse number, while macromolecule transport is controlled by pulse duration [25]. Previous studies revealed that DNA transfer through the plasma membrane is a complex process that includes two steps. The first step is fast and creates DNA–membrane interaction [26]. The second step is translocating the DNA by endocytosis, which occurs several minutes later [27,28], and finally, the subsequent diffusion into the nucleus. Setting 2 involves two electroporation parameters, the poring, a short high-voltage step to induce pores (poring), and a long low-voltage step to transfer macromolecules (Table 1). In setting 2, poring parameters included four unipolar electric pulses of 40 volts for 3.5 ms, which form the plasma membrane pores. The transfer parameters included five bipolar electric pulses of 5 volts for 50 ms, which are designed to expedite the translocation of the RNPs to the cytoplasm by electrophoretic displacement. Electric pulses characterized by low voltage and long duration boosted DNA transfer in vivo [29,30]. Low voltage exerts the required forces to bring charged molecules to the membrane [31,32]. Furthermore, applying pulses with different polarity enhance the multi-directional transfer of the negatively charged RNPs to the plasma membrane. Further investigations are needed to clarify the RNPs translocation process to the zygotic cytoplasm.

The decrease in blastocyst rate and mutation percentage of setting 3 when compared to setting 2 could be associated with a higher number of pulses in setting 3 (6 bipolar pulses of 30 volts for 3 ms). Additionally, short-length pulses can disturb DNA transmembrane transport [25] and may have a similar effect on RNPs.

The high efficiency of CRISPR/Cas9 RNP electroporation for introducing a biallelic mutation in sheep embryos was additionally confirmed by targeting the PDX1 gene with the optimized conditions. The mutation rate was 82.2% ± 2%, with a biallelic mutation rate of 81% ± 5%. In a previous study, the same sgRNAs produced 46% and 20% biallelic mutations when microinjected into sheep MII oocytes and zygotes, respectively [33].

Finally, electroporation efficiency for delivering the CRISPR-Cas9 system was further confirmed by targeting goat OTX2 using a dual gRNA approach resulting in a long deletion of 1126 bp in 33 out of 41 zygotes (80.5% ± 5%) with 28 embryos (85% ± 6%) presenting biallelic mutations. These results confirm the high efficiency of both the CRISPR-Cas9 and electroporation to introduce long deletions.

To the best of our knowledge, this is the first report of a successful introduction of the CRISPR-Cas9 system into the intact zygote of sheep and goats by electroporation to generate knock-out blastocysts.

## 4. Materials and Methods

### 4.1. Experimental Design

We designed three experiments to examine the efficiency of electroporation in delivering the CRISPR-Cas9 into the small ruminants’ zygotes, each with three replicates. In experiment 1, we targeted sheep SOCS2 locus and tested three different electroporation settings at four different developmental times: MII oocytes and zygotes at 6, 8, and 10 h after fertilization. We compared the efficiency of gene editing with microinjection at two times: MII oocytes and 6–8 h after fertilization. In experiment 2, we selected the most efficient electroporation conditions to target the sheep PDX1 locus to confirm the electroporation efficiency at another sheep locus. In experiment 3, we targeted the goat OTX2 locus to confirm the electroporation efficiency in targeting another small ruminant species.

### 4.2. sgRNAs

**Figure 6:**
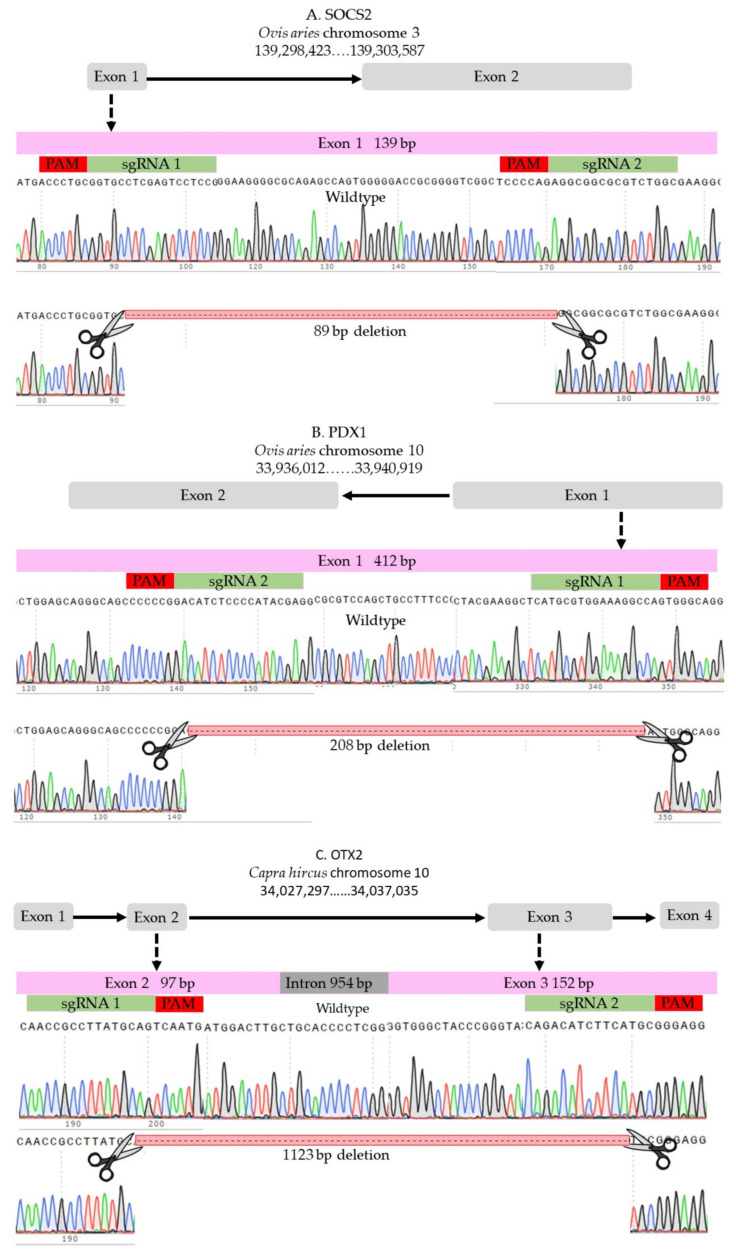
sgRNAs. (**A**) SOCS2 in sheep. (**B**) PDX1 in sheep. (**C**) OTX2 in goat.

Two sgRNAs were designed to target each locus using the online designing tools (http://www.e-crisp.org, accessed on 28 August 2019) and (http://chopchop.cpu.uib.no, accessed on 28 August 2019) and were ordered from Synthego (Synthego Corporation, Redwood City, CA, USA). The selected sgRNAs fulfilled stringent criteria of efficiency and specificity. For ovine SOCS2 locus, two sgRNAs were selected to target the beginning and the end of exon 1 (Figure 6A, and Supplementary Table S1); the expected deletion was 85 bp. For ovine PDX1, we used two sgRNAs that were successfully used in a previous study [33]; the expected deletion was 208 bp of exon 1 (Figure 6B and Supplementary Table S1). The caprine OTX2 gene has four exons, and we selected two sgRNAs to induce a long deletion of 1123 bp to disrupt exons 2 and 3 (Figure 1C and Supplementary Table S1).

### 4.3. Gametes

#### 4.3.1. Cumulus Oocytes Complexes (COCs)

Sheep and goat ovaries were collected from a local abattoir (Superior Farms, Sacramento, CA, USA) and transported to the laboratory in warm saline. COCs (1–8 mm) were aspirated by using a 21 G butterfly needle using vacuum pressure. All work was performed in a warm and disinfected workstation.

#### 4.3.2. Semen

Fresh ram semen was collected before in vitro fertilization and extended using Andromed (Minitube, Tiefenbach, Germany) at a 1:1 ratio. Frozen goat semen was provided by B & D Genetics.

### 4.4. In Vitro Embryo Production

All work was performed in a warm and disinfected workstation. All media were pre-warmed before use. Incubation work was performed at 38.5 °C in a humified environment at 5% O_2_, 5% CO_2,_ and 90% N_2_.

### 4.5. In Vitro Maturation

Suitable-quality COCs with several cumulus cells and homogeneous cytoplasm layers were selected for in vitro maturation. First, COCs were washed five times with warm washing media and then transferred in groups of 50 to 400 µL of maturation medium (BO-IVM, IVF Bioscience, Falmouth, UK) and incubated for 22–24 h.

### 4.6. In Vitro Fertilization

The expanded COCs were washed three times with warm and pre-equilibrated fertilization media (BO-IVF, IVF Bioscience) and transferred in groups of 50 to 400 µL of fertilization media. Semen was washed twice by centrifugation at 328× *g* for 5 min with 2 mL of semen preparation media (BO-Semen Prep, IVF Bioscience). Sperm concentration was adjusted with fertilization media to 2 × 10^6^/mL and added to COCs. The final fertilization drop volume was 500 µL. When the microinjection or electroporation was performed at the MII oocyte, the cumulus was stripped, and the stripped oocytes were incubated with sperms in the presence of 10 COCs.

### 4.7. In Vitro Culture

Fertilized COCs were stripped by vortexing for 3 min in SOF-Hepes media, washed five times, and transferred in a group of 50 to 500 µL of culture media (BO-iVC, IVF Bioscience), covered with 400 µL of mineral oil, and incubated for seven days.

### 4.8. Ribonucleoprotein (RNP) Preparation

All work of RNA was carried out in a disinfected and RNA-free workstation at room temperature. Before each delivery session, the sgRNA was mixed with Cas9 protein (PNA Bio, Thousand Oaks, CA, USA) at a 1:2 concentration ratio and incubated on ice for 10 min. The final concentration of each sgRNA was 40 ng/µL.

### 4.9. Microinjection

Microinjection was performed in a micromanipulation station that consisted of micromanipulators (Narishige, Tokyo, Japan) attached to an inverted microscope (Nikon, Tokyo, Japan) and two hydraulic oil microinjectors (Eppendorf, Hamburg, Germany). Denuded zygotes and MII oocytes were washed three times with SOF-Hepes and transferred in a group of 25 to 50 µL drops of SOF-Hepes covered with mineral oil. To confirm getting into the cytoplasm, a zygote or MII oocyte was secured by a holding pipette (150–180 µm outside diameter), and the zona pellucida was drilled by a laser system (Saturn 5, RI, Falmouth, UK), followed by aspirating part of the cytoplasm through the injection needle (5–7 µm) via applying an appropriate negative pressure [34]. Finally, positive pressure was applied softly to force out approximately 6 pL of the RNPs into the cytoplasm.

### 4.10. Electroporation

Electroporation was performed using NEPA21 Super Electroporator and Nepa Electroporation Cuvettes 1 mm gap (EC-001) (Nepagene, Ichikawa-City, Japan). All work was performed in a disinfected and RNA-free workstation. Denuded MII oocytes/zygotes were washed three times with SOF-Hepes, three times with warm Opti-MEM reduced serum medium (Thermo Fisher Scientific #31985062), then moved to a new drop of Opti-MEM and mixed with RNPs to bring the volume to 20 µL and loaded to the bottom of the cuvette. The electroporation process was conducted in one of three different settings (Table 1). After electroporation, the cuvette was washed several times with SOF-Hepes, and the recovered MII oocytes/zygotes were washed three times with SOF-Hepes and one time with BO-IVC and moved to pre-warmed and pre-equilibrated BO-IVC for culture

### 4.11. Indel Detection

**Figure 7:**
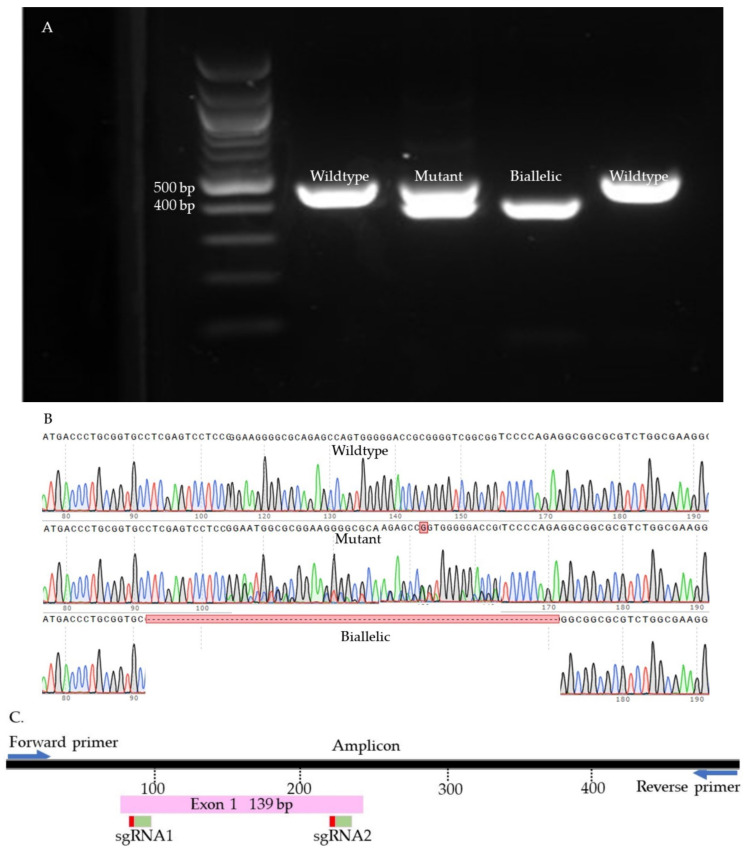
SOCS2 knock-out samples. (**A**). Gel electrophoresis. (**B**). Sanger sequences (**C**). Map of the targeted site.

**Figure 8:**
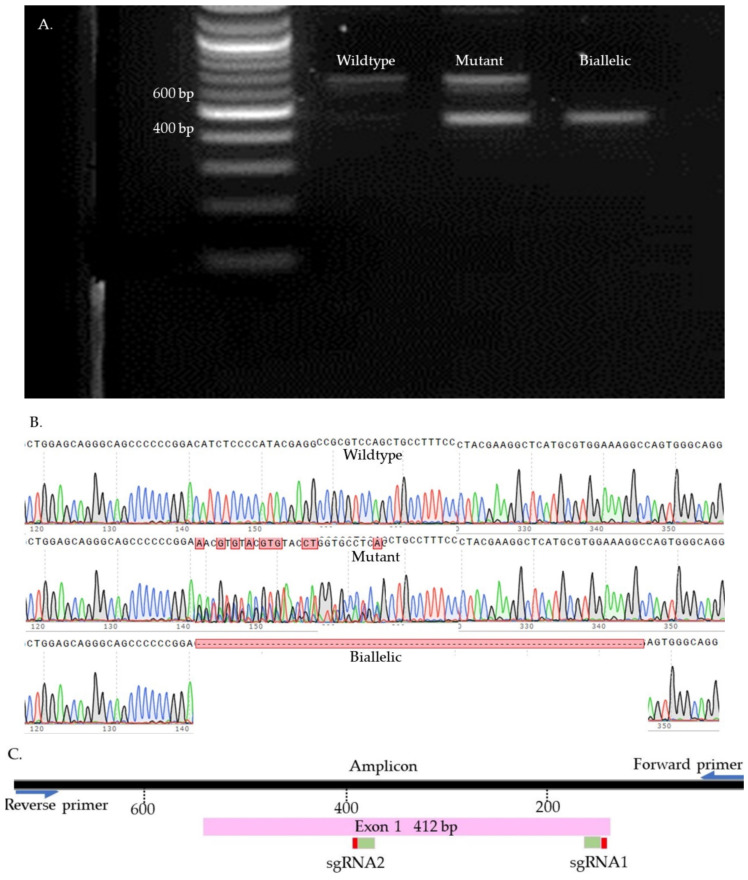
PDX1 knock-out samples. (**A**). Gel electrophoresis. (**B**). Sanger sequences (**C**). Map of the targeted site.

**Figure 9:**
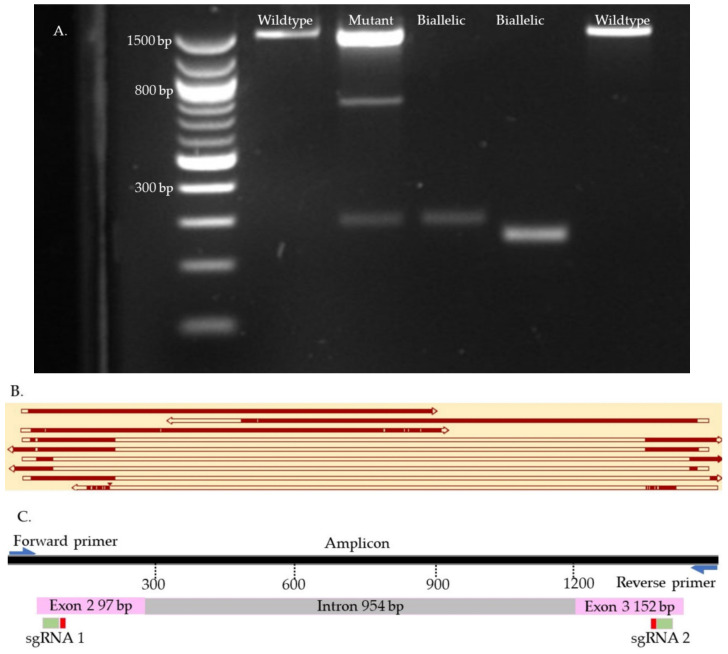
OTX2 knock-out samples. (**A**). Gel electrophoresis. (**B**). Map of some knock-outs samples. (**C**). Map of the targeted site. The first two top samples are wild type. The second sample is mutant. The six bottom samples are biallelic. The gaps in the sequences represent reading errors or deletions.

We used Sanger sequencing and Tracking of Indels by Decomposition (TIDE) to evaluate the editing efficiency [35]. Briefly, on day seven, blastocysts were picked up and placed individually in 0.2 mL PCR tubes containing 10 µL of DNA extraction buffer (Epicentre), centrifuged for 10 s, and incubated at 65 °C for 6 min and 98 °C for 2 min. Extracted DNA was mixed with 10 µL of GoTaq Hot Start Green Master Mix (Promega, Madison, WI, USA) and 0.8 µL of previously designed and tested primers (Supplementary Table S2). The amplification process was performed in two runs of PCR. The condition of each run was 95 °C for 5 min, 35 cycles of 95 °C for 30 s, 58 °C for 30 s, 72 °C for 45 s, and 72 °C for 10 min. To visualize the size of the amplicon, the PCR product was separated by 2% gel electrophoresis and compared to a control amplicon. DNA bands were extracted from the gel and purified by QIAquick Gel extraction kit (QIAGEN) and sent for Sanger sequencing (Genewiz, Shinagawa City, Tokyo). To detect the indels, first, each amplicon sequence was aligned to a control amplicon sequence using SnapGene software (Dotmatics, San Diego, CA, USA) to visualize the modifications. Sequences were further analyzed using the Inference of CRISPR Editing (ICE) online tool (https://ice.synthego.com/, accessed on 28 August 2019) to estimate the editing efficiency. The samples were classified as either wild-type or mutant (Figure 7, Figure 8, Figure 9 and Supplementary Figure S1). The wild-type samples have the same amplicon size and sequence as the control. The mutant samples were considered biallelic when they had a smaller band in the gel compared to the control, deletion in sequence, and there were no wild-type alleles. When the wild-type sequences were presented in the mutant samples, the samples were considered mutants.

### 4.12. Statistical Analysis

The percentages of blastocysts, mutation, and biallelic mutations were analyzed by one-way ANOVA. When there was a significant difference at *p* < 0.05, a Tukey range test was employed to compare the means.

## Figures and Tables

**Table 1:** Electroporation parameters.

Setting	Electroporation Parameters						
	Pulse Type	Voltages (V)	Pulse Length (ms)	Pulse Interval (ms)	Pulses Number	Decay Rate (%)	Polarity
1	Poring	40	3.5	50	4	10	+
2	Poring	40	3.5	50	4	10	+
	Transfer	5	50	50	5	40	+/−
3	Poring	30	3	100	6	0	+/−

## Supplementary Materials

The supporting information can be downloaded at: https://www.mdpi.com/article/10.3390/ijms231810218/s1.

## References

[1] Kalds P., Gao Y., Zhou S., Cai B., Huang X., Wang X., Chen Y. (2020). Redesigning small ruminant genomes with CRISPR toolkit: Overview and perspectives. Theriogenology.

[2] Jiang F., Doudna J.A. (2017). CRISPR–Cas9 Structures and Mechanisms. Annu. Rev. Biophys..

[3] Charpentier E., Doudna J.A. (2013). Biotechnology: Rewriting a genome. Nature.

[4] Liu Z., Liao Z., Chen Y., Han L., Yin Q., Xiao H. (2020). Application of Various Delivery Methods for CRISPR/dCas9. Mol. Biotechnol..

[5] Crispo M., Mulet A.P., Tesson L., Barrera N., Cuadro F., dos Santos-Neto P.C., Nguyen T.H., Crénéguy A., Brusselle L., Anegón I. (2015). Efficient Generation of Myostatin Knock-Out Sheep Using CRISPR/Cas9 Technology and Microinjection into Zygotes. PLoS ONE.

[6] Li X., Hao F., Hu X., Wang H., Dai B., Wang X., Liang H., Cang M., Liu D. (2019). Generation of Tβ4 knock-in Cashmere goat using CRISPR/Cas9. Int. J. Biol. Sci..

[7] Czernik M., Anzalone D.A., Palazzese L., Oikawa M., Loi P. (2019). Somatic cell nuclear transfer: Failures, successes and the challenges ahead. Int. J. Dev. Biol..

[8] Tan W., Proudfoot C., Lillico S.G., Whitelaw C.B.A. (2016). Gene targeting, genome editing: From Dolly to editors. Transgenic Res..

[9] Grabarek J.B., Plusa B., Glover D.M., Zernicka-Goetz M. (2002). Efficient delivery of dsRNA into zona-enclosed mouse oocytes and preimplantation embryos by electroporation. Genesis.

[10] Kaneko T., Sakuma T., Yamamoto T., Mashimo T. (2014). Simple knockout by electroporation of engineered endonucleases into intact rat embryos. Sci. Rep..

[11] Qin W., Dion S.L., Kutny P.M., Zhang Y., Cheng A.W., Jillette N.L., Malhotra A., Geurts A.M., Chen Y.-G., Wang H. (2015). Efficient CRISPR/Cas9-Mediated Genome Editing in Mice by Zygote Electroporation of Nuclease. Genetics.

[12] Tanihara F., Hirata M., Nguyen N.T., Le Q.A., Hirano T., Takemoto T., Nakai M., Fuchimoto D.-I., Otoi T. (2018). Generation of a TP53-modified porcine cancer model by CRISPR/Cas9-mediated gene modification in porcine zygotes via electroporation. PLoS ONE.

[13] Ciccarelli M., Giassetti M.I., Miao D., Oatley M.J., Robbins C., Lopez-Biladeau B., Waqas M.S., Tibary A., Whitelaw B., Lillico S. (2020). Donor-derived spermatogenesis following stem cell transplantation in sterile NANOS2 knockout males. Proc. Natl. Acad. Sci. USA.

[14] Flores-Morales A., Greenhalgh C.J., Norstedt G., Rico-Bautista E. (2006). Negative Regulation of Growth Hormone Receptor Signaling. Mol. Endocrinol..

[15] Offield M.F., Jetton T.L., Labosky P.A., Ray M., Stein R.W., Magnuson M.A., Hogan B.L., Wright C.V. (1996). PDX-1 is required for pancreatic outgrowth and differentiation of the rostral duodenum. Development.

[16] Mortensen A.H., Schade V., Lamonerie T., Camper S.A. (2014). Deletion of OTX2 in neural ectoderm delays anterior pituitary development. Hum. Mol. Genet..

[17] Son R.S., Smith K.C., Gowrishankar T.R., Vernier P.T., Weaver J.C. (2014). Basic Features of a Cell Electroporation Model: Illustrative Behavior for Two Very Different Pulses. J. Membr. Biol..

[18] Casciola M., Kasimova M.A., Rems L., Zullino S., Apollonio F., Tarek M. (2016). Properties of lipid electropores I: Molecular dynamics simulations of stabilized pores by constant charge imbalance. Bioelectrochemistry.

[19] Kloc M., Ghobrial R.M., Borsuk E., Kubiak J.Z. (2012). Polarity and Asymmetry During Mouse Oogenesis and Oocyte Maturation. Results Probl. Cell Differ..

[20] Hashimoto M., Yamashita Y., Takemoto T. (2016). Electroporation of Cas9 protein/sgRNA into early pronuclear zygotes generates non-mosaic mutants in the mouse. Dev. Biol..

[21] Benov L.C., Antonov P.A., Ribarov S.R. (1994). Oxidative damage of the membrane lipids after electroporation. Gen. Physiol. Biophys..

[22] Chen W., Zhongsheng Z., Lee R.C. (2006). Supramembrane potential-induced electroconformational changes in sodium channel proteins: A potential mechanism involved in electric injury. Burns.

[23] Khan A.S., Smith L.C., Abruzzese R.V., Cummings K.K., Pope M.A., Brown P.A., Draghia-Akli R. (2003). Optimization of Electroporation Parameters for the Intramuscular Delivery of Plasmids in Pigs. DNA Cell Biol..

[24] Heller R., Shi G. (2021). Controlled Delivery of Plasmid DNA to Melanoma Tumors by Gene Electrotransfer. Methods Mol. Biol..

[25] Rols M.-P., Teissie J. (1998). Electropermeabilization of Mammalian Cells to Macromolecules: Control by Pulse Duration. Biophys. J..

[26] Golzio M., Teissié J., Rols M.-P. (2002). Direct visualization at the single-cell level of electrically mediated gene delivery. Proc. Natl. Acad. Sci. USA.

[27] Rosazza C., Escoffre J.-M., Zumbusch A., Rols M.-P. (2011). The Actin Cytoskeleton Has an Active Role in the Electrotransfer of Plasmid DNA in Mammalian Cells. Mol. Ther..

[28] Wu M., Yuan F. (2011). Membrane Binding of Plasmid DNA and Endocytic Pathways Are Involved in Electrotransfection of Mammalian Cells. PLoS ONE.

[29] Cemazar M., Golzio M., Sersa G., Hojman P., Kranjc S., Mesojednik S., Rols M.-P., Teissie J. (2009). Control by pulse parameters of DNA electrotransfer into solid tumors in mice. Gene Ther..

[30] Goto T., Nishi T., Tamura T., Dev S.B., Takeshima H., Kochi M., Yoshizato K., Kuratsu J.-I., Sakata T., Hofmann G.A. (2000). Highly efficient electro-gene therapy of solid tumor by using an expression plasmid for the herpes simplex virus thymidine kinase gene. Proc. Natl. Acad. Sci. USA.

[31] Kandušer M., Miklavčič D., Pavlin M. (2009). Mechanisms involved in gene electrotransfer using high- and low-voltage pulses—An in vitro study. Bioelectrochemistry.

[32] Haberl S., Kandušer M., Flisar K., Hodžić D., Bregar V.B., Miklavčič D., Escoffre J.-M., Rols M.-P., Pavlin M. (2013). Effect of different parameters used for in vitro gene electrotransfer on gene expression efficiency, cell viability and visu-alization of plasmid DNA at the membrane level. J. Gene Med..

[33] Vilarino M., Rashid S.T., Suchy F.P., McNabb B.R., Van Der Meulen T., Fine E.J., Ahsan S.D., Mursaliyev N., Sebastiano V., Diab S.S. (2017). CRISPR/Cas9 microinjection in oocytes disables pancreas development in sheep. Sci. Rep..

[34] Bogliotti Y.S., Vilarino M., Ross P.J. (2016). Laser-assisted Cytoplasmic Microinjection in Livestock Zygotes. J. Vis. Exp..

[35] Brinkman E.K., Chen T., Amendola M., Van Steensel B. (2014). Easy quantitative assessment of genome editing by sequence trace decomposition. Nucleic Acids Res..

