# Supplementary material for: Single-Step Genome Editing of Small Ruminant Embryos by Electroporation
Source: Int J Mol Sci. 2022 Sep 6;23(18):10218. doi: 10.3390/ijms231810218 (PMC9499182; doi:10.3390/ijms231810218)
Supplement: Supplementary file 1 [file ijms-23-10218-s001.zip › ijms-1705388-supplementary.pdf]

Supplementary Materials

Table S1. sgRNA sequences.

No	Target	Species	sgRNA Sequence
1	SOCS2	Ovis aries	sgRNA1 GGAGGACUCGAGGCACCGC
			sgRNA2 UCGCCAGACGCGCCGCCUCU
2	PDX1	Ovis aries	sgRNA1 UCAUGCGUGGAAAGGCCAGU
			sgRNA2 UCGUAUGGGGAGAUGUCCGG
3	OTX2	Capra hircus	sgRNA1 AACCGCCUUAUGCAGUCAAU
			sgRNA2 AGACAUCUUCAUGCGGGAGG

Table S2. Primers sequences.

No	Target	Species	Primers sequence	Amplicon Size bp
1	SOCS2	Ovis aries	Forward AGGGAGTGGTTTTGGGGTTC	479
			Reverse AGCGCCCTAAGAGTCGATTT	
2	PDX1	Ovis aries	Forward AGGAAGAGCGCTGAG-TAGAAAG	652
			Reverse CATGAACCGCGAGGAGCAGTA	
3	OTX2	Capra hircus	Forward GTGGAACGTGAAAGAGCTGC	1519
			Reverse TCCTTTGCTCGTTTCCTGGAG	

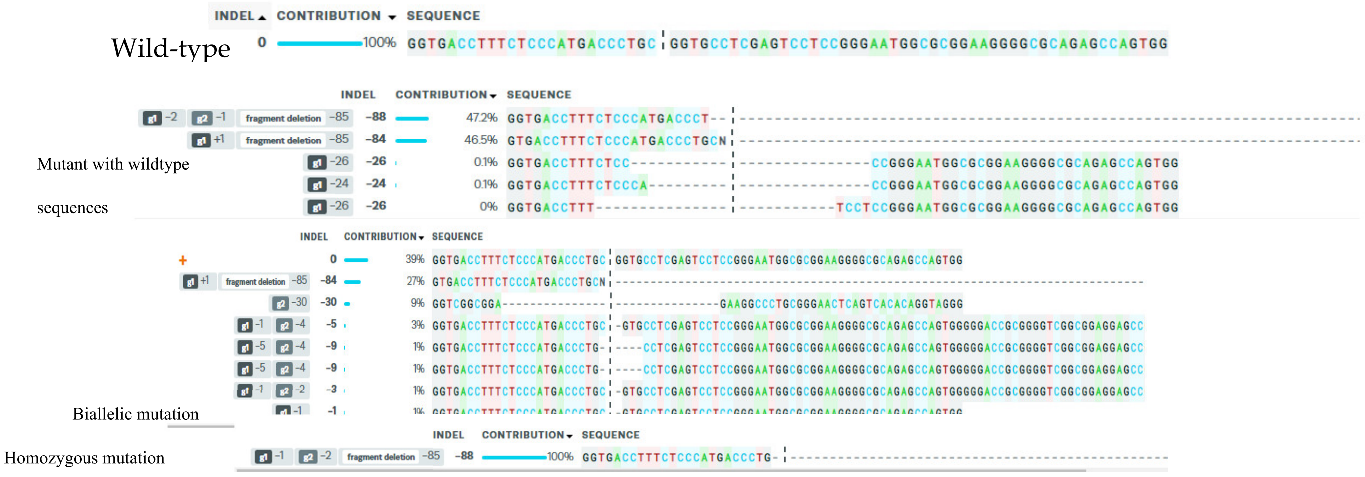

Supplementary Figure S1. The Inference of CRISPR Editing (ICE) analysis of some samples.
